# What Health Service Provider Factors Are Associated with Low Delivery of HIV Testing to Children with Acute Malnutrition in Dowa District of Malawi?

**DOI:** 10.1371/journal.pone.0123021

**Published:** 2015-05-01

**Authors:** Lusungu Chitete, Thandi Puoane

**Affiliations:** School of Public Health, University of the Western Cape, Cape Town, Western Cape Province, Republic of South Africa; Faculty of Medicine, AUSTRALIA

## Abstract

**Background:**

The Community-based Management of Acute Malnutrition is the national program for treating acute malnutrition in Malawi. Under this program’s guidelines all children enrolled should undergo an HIV test, so that those infected can receive appropriate treatment and care. However, the national data of 2012 shows a low delivery of testing. Prior studies have investigated client-related factors affecting uptake of HIV testing in Community-based Management of Acute Malnutrition program. Lacking is the information on the service provider factors that are associated with the delivery of testing. This study investigated service provider factors that affect delivery of HIV testing among children enrolled in the program and explored ways in which this could be improved.

**Methods:**

A descriptive study that used qualitative methods of data collection. Client registers were reviewed to obtain the number of children enrolled in Community-based Management of Acute Malnutrition and the number of children who were tested for HIV over a 12-month period. In-depth interviews were conducted with Community-based Management of Acute Malnutrition and HIV Testing and Counselling focal persons to investigate factors affecting HIV test delivery. Descriptive statistics were used to analyze data from client registers. Information from interviews was analyzed using a thematic approach.

**Results:**

Quantitative data revealed that 1738 (58%) of 2981 children enrolled in Community-based Management of Acute Malnutrition were tested for HIV. From in-depth interviews four themes emerged, that is, lack of resources for HIV tests; shortage of staff skilled in HIV testing and counseling; lack of commitment among staff in referring children for HIV testing; and inadequately trained staff.

**Conclusion:**

There is a need for a functioning health system to help reduce child mortality resulting from HIV related conditions.

## Introduction

Acute malnutrition and HIV are major public health problems in Malawi. Nearly half (47%) of Malawian children are stunted; 13 percent are underweight and 4 percent are wasted. The country has an estimated HIV prevalence of 11 percent in the 15–49 year age group [1], with about a million people living with HIV [2].

There were 230,000 children newly infected with HIV in Sub Saharan Africa in 2012, mostly through transmission from mother to child, accounting for 14% of all new infections in the region [3]. In HIV-exposed babies, HIV infection affects growth and nutrition status starting in the peri-natal stages, frequently resulting in low birth weight. After birth, if not adequately treated, it may lead to underweight and wasting or even death unless remedial interventions such as anti-retroviral therapy (ART) are administered [4]. Studies show that HIV-infected children with severe acute malnutrition are three times more likely to die, particularly in the absence of ART, than uninfected children [5]. HIV infection affects appetite, absorption of nutrients, altered metabolism and may lead to secondary infections among people living with HIV [6].

Community-based management of acute malnutrition (CMAM) is the national program for addressing severe and moderate acute malnutrition in Malawi. The CMAM has three treatment components: Nutrition Rehabilitation Units (NRU) where severely malnourished children with medical complications and poor appetite are treated as in-patients; Outpatient Therapeutic Program (OTP) where severely malnourished children without complications and with good appetite are treated as out-patients; and Supplementary Feeding Program (SFP) where children with moderate acute malnutrition are treated as out-patients.

Under this program’s guidelines [7], all children suffering from acute malnutrition should be tested for HIV, so that those diagnosed HIV-positive can be referred to appropriate care. However, national CMAM data shows that only a fraction of children are tested for HIV. For example, of the 64,677 children newly admitted in CMAM in 2012, only 16,672 were tested for HIV, a delivery of 26% [8]. The CMAM admits children aged between 6 months and 11 years. Typically children are tested using rapid antibody test, as opposed to DNA Polymerase Chain Reaction (PCR) which is recommended for children younger than 6 weeks. However, in the absence of DNA PCR, antibody test is still performed for children under 6 weeks. Children aged 12 or below are tested only with consent of a parent or guardian; while those aged 13 years and above can consent to an HIV test [9].

When a child presents at a CMAM clinic they first undergo anthropometric measurements. Those that are diagnosed with acute malnutrition are enrolled by having their bio-data recorded in registers. A health talk is given to all clients on malnutrition, HIV and general child care; after which they are referred to HIV Testing and Counselling (HTC) and clinical review. Children who are found to be HIV positive may then be prescribed ART depending on their condition. Also, if a caretaker is a pregnant woman and is found to be HIV-positive, she is enrolled in prevention of mother-to-child transmission of HIV (PMTCT).

Prior studies that have investigated factors which affect uptake of HTC amongst CMAM clients have focused on patient or demand related factors [10] [11]. No studies have investigated how service provider factors affect the delivery of HIV testing. The current study investigated service provider factors which need to be addressed for delivery of HIV testing to be improved.

## Materials and Methods

The study was undertaken in Dowa, a rural district located in the central region of Malawi, neighboring Lilongwe, the capital city of Malawi. Dowa district has a population of 559,950 people, of which 274,674 are male and 285,276 are female; 97,372 are children under the age of five; 216,943 are aged between 5 and 19; and 245, 635 are aged above 20 years[12]. Administratively, the district is divided into 7 traditional authorities, which are further sub-divided into group-villages and then villages as the smallest administrative unit.

The majority of the people of Dowa district subsist by small-scale non-commercial farming. Mobility within the district, including access to health facilities, is typically by foot, bicycles or ox-cart through dirt roads.

Dowa district was purposely selected because among the four districts neighboring Lilongwe it has the highest coverage of CMAM services, particularly OTP and SFP.

The study population included 16 government-run health facilities, which also have HTC services in Dowa district. As standard protocol all pregnant women, including caretakers of children enrolled in CMAM, are offered HTC together with the malnourished child in their care. At health centres, HTC is conducted by health surveillance assistants (HSA) who undergo generic Ministry of Health training which is repeated as and when new testing protocols are introduced. Pregnant women found to be HIV-positive are then enrolled in PMTCT. All facilities in Dowa District have PMTCT which is administered by clinicians and nurses. Since the study sought to investigate delivery of HIV testing in CMAM, only sites that had both HTC and CMAM services were invited to participate in the study. Of these 16 sites, 6 sites were randomly sampled, representing 38% of the government facilities in the district.

Interviewees were purposively selected by virtue of being focal persons of either CMAM or HTC. All 6 sampled facilities have one focal person for CMAM and another for HTC. The focal persons were selected because they possess knowledge on the overall performance of the programs they oversaw within the health facility. As standard practice, focal persons held monthly meetings with all their cadre to review program progress and challenges. This forum, among others, provided them with views of other staff which were vital for the purpose of this study. At each of the 6 sampled health facilities, two focal persons were selected; making a total of 12 facility-based staff. Overall, a total of 14 interviewees (two staff at the district health office and 12 facility-level health workers) participated in the study. All focal persons selected accepted the interview.

## Study Design and Data Collection

A descriptive study utilized qualitative methods of data collection. Data was collected between 27 March and 4 April 2013. To determine the number of children enrolled in CMAM who were tested for HIV, data was extracted from CMAM client registers covering the previous 12 months (January–December 2012). In total six registers were reviewed; one at each of the sampled sites. For each month, the number of clients enrolled in CMAM was tallied against the number tested for HIV. Percentages were computed for each site.

In-depth interviews were conducted with the CMAM and HTC focal persons to explore factors that affected delivery of HIV testing, as well as ways to improve delivery. Interview questions were pretested on two health workers (one focal person of CMAM and another of HTC) in a facility that had not been sampled in the same district. Open-ended questions were asked such as: “Some of your clients are not tested for HIV; what would you say are the factors behind that?” Where respondents’ answers were unclear, we sought clarification or probed for more information. Interviews were tape-recorded and notes were taken. Interviews were conducted in a combination of English and Chichewa, the former being the official language and the latter the most widely spoken language in Malawi. Interviews were continued until saturation. All interviews were conducted by the first author who is not associated with the health facilities or the district. Interviews were conducted in a private room and no other staff members were involved. One interview took an average 45 minutes

After the analysis of quantitative data was completed it became clear that we needed to explore the reasons for the differences in delivery of HIV testing between the centers that performed well and those that performed poorly in HIV testing. Two health workers were purposely selected from the facilities that had achieved between 80% and 100% HIV testing delivery and two from facilities with the lowest delivery. They were invited for interviews to explore reason for the differences in performance.

## Ethics Statement

Ethics approval for the study was obtained from the University of the Western Cape Ethics Committee and from Malawi’s National Health Sciences Research Committee.

All participants in the study provided prior written informed consent. The consent procedure was approved by the University of the Western Cape Ethics Committee and Malawi’s National Health Sciences Research Committee. The signed consent forms are on authors’ hard copy file.

Data from CMAM client register was anonymized. The use of this anonymized data was approved by the two ethics committees as part of the entire study design and as such did not require separate approval from database managers of client registers.

## Data Analysis

Thematic analysis was conducted by the authors to analyse qualitative data from in-depth interviews. Data was categorized and summarized into common emerging themes in terms of factors affecting delivery of HIV testing.

To ensure data credibility and trustworthiness data, information provided by one respondent was checked against information given by another respondent in the same facility. Information gathered at facilities was also cross-checked with district focal persons. All interview data were written down in a notebook and tape-recorded as back-up. At the end of each interview, notes were summarized and dictated back to the respondent to check if the information was an accurate reflection of the interview.

## Results, Discussion and Conclusions

### Demographic Characteristics of Study Participants

Table 1 displays the demographic characteristics of participants. A total of 14 health workers participated in the study. Their ages ranged between 30 to 50 years. Ten participants were males and 4 were females. Twelve participants were health surveillance assistants; one was a nutritionist and another was a laboratory technician. Twelve participants had obtained O-level and two were educated above O-level.

**Table 1:** Participant demographic characteristics (n = 14).

Characteristic	Male	Female	Total
Age:			
30–40	5	3	8
41–50	5	1	6
Education:			
O Level	9	3	12
Above O level	1	1	2
Cadre:			
Health Surveillance Assistant	9	3	12
Nutritionist	0	1	1
Laboratory technician	1	0	1

### Quantitative data

Table 2 shows that of the 2981 children newly enrolled into CMAM during the study period the highest number that was tested was 1738, representing a delivery of 58%.

**Table 2:** Percentage uptake of HIV testing in health facilities (n = 6).

Service type	Number newly enrolled	Number HIV-tested	Percentage tested
OTP Site 1	89	82	92%
SFP Site 1	360	324	90%
OTP Site 2	90	77	86%
SFP Site 2	386	328	85%
OTP Site 3	92	57	62%
SFP Site 3	379	216	57%
OTP Site 4	101	45	45%
SFP Site 4	400	182	46%
OTP Site 5	87	22	25%
SFP Site 5	386	80	21%
NRU Site 6	123	123	100%
OTP Site 6	139	63	45%
SFP Site 6	349	139	40%
	2981 (Total)	1738 (Total)	58% (Average)

There were marked differences in percentage of children tested between facilities. For example, NRU at Dowa District Hospital had 100% of children tested for HIV over the study period. Two other facilities tested over 80% of children. The rest of the facilities (three) achieved much lower percentage of children tested, ranging from 21 percent to 62 percent.

### Qualitative findings

From qualitative data the following themes emerged:

#### Lack of resources for HIV testing

The main resource affecting delivery of HIV testing was test kits. All sites but one reported intermittent supply of HIV testing kits throughout the period of the study. At the time of the study, two sites were found to have curtailed HIV testing for all clients except pregnant women for the purpose of PMTCT. The following quote from a respondent exemplifies this finding:

“…for example, right now there is nothing we can do. We do not have enough test kits, so the few that we have we are using them to test pregnant women”

The district HTC focal person added that poor coordination within health facilities in placing of orders as also contributing to shortage of test kits. He said:

“I noticed that when orders are placed for test kits some departments in health facilities are not consulted, leading to insufficient supplies provided to them”

#### Shortage of staff skilled in HIV testing and counseling

This factor was reported in 4 of the 6 study sites. Where the challenge was reported (staffed by an average 3 counselors per site), it was found that HTC staff are not full-time counselors but have other duties like the rest of their cadre, HSA. As such, they may be absent from the testing facility in parts of the day or days of the week assigned to them by roster. When this happens, clients including those referred from CMAM cannot be tested. An HTC counselor said that:

“The thing is that we are not full-time counselors; we are health surveillance assistants like everyone else. So, we have to juggle HIV testing and other duties in the catchment area allocated to us”

#### Lack of commitment among staff in referring children for HIV testing

This challenge is two-fold: on one hand, not all children are referred to HIV testing at entry into CMAM; and on the other, those not tested at entry are not fully followed up in subsequent visits. This was reported in all but two sites. The reasons behind non-referral were mainly lack of motivation to or/and appreciation on the part of CMAM staff of importance of referring malnourished children to HIV testing. One CMAM focal person said:

“What I think is that a lot of our staff do not think that it is such an important thing to refer children for testing. That is where the problem lies”

The lack of diligence in referring children to testing was attributed to another factor, which is that at the time CMAM staff were last trained (four years earlier) there had been little emphasis, if any, on such referral.

One health worker, however, explained as follows:

“I have seen clients that were initially not referred that when they are followed up with counselling, most of them eventually accept the test”

#### Inadequately trained staff

The study found that all 94 health workers involved in CMAM had been trained in management of acute malnutrition. However, the majority had last been trained about 4 years earlier.

All 6 facility-based CMAM focal persons interviewed reported that staff was generally in need of more up-to-date refresher training. Notably, respondents cited lack of skills among CMAM staff in motivating or counseling mothers to have their children tested. One focal person of CMAM said:

“Most of the staff does not have skills to convince clients to go and test. As you know, CMAM staff is not trained in counseling. I think that if staff received training in counseling, we would be able to refer more clients to HIV testing”.

#### Factors that explained the differences in performance among centers

Qualitative data revealed that in the facilities that managed to test 80% to100% of children, health workers devoted deliberate efforts towards intensive and repeated follow-up counselling of clients until they all accept to be tested for HIV. This intensive counselling is peculiar to the NRU because it enrolls the most of severe cases of acute malnutrition, i.e. children who are severely malnourished, have medical complications and low appetite. As such, the staff deemed it as imperative that all children are tested so that if they are HIV-positive they should be put on the correct treatment to prevent unnecessary death.

Similarly, in the facilities that had managed to test over 80% of children, the repeated follow-up counselling was found to be the reason behind such success.

On the other hand, in the facilities where the percentage of children was lowest (40% to 62%) there was less follow-up client counselling to test for HIV. Clients were approached once and, if they did not consent for testing, they were mostly left alone due to lack of diligence on the part of the health workers.

### How delivery of HIV testing could be increased

Respondents suggested that delivery of HIV testing could be improved through the following:

#### Ensure sufficient and uninterrupted supply of HIV test kits

Five of the 6 study sites recommended this as a means to increase delivery of testing among children enrolled in CMAM. The one site that did not cite this measure also did not report a challenge is the supply of test kits.

#### Deploy more staff in HTC

In sites which cited inadequate HTC staff as negatively affecting delivery of HIV testing (5 of the 6 sites in study) interviewees suggested increasing the number of HTC counselors. These sites reported overwhelming workload for counselors who on one hand are inadequate in numbers and on the other had to perform other duties required of them as health surveillance assistants.

#### Provide training to CMAM staff in counseling

In 4 of the 6 sites, CMAM focal persons reported that more know-how amongst staff in counseling may help to motivate clients and increase acceptance of testing. This training need not be full HTC training such as is provided to certified counselors.

#### Orient CMAM staff on importance of referral to HIV testing

In 4 of the 6 sites studied it was contended that more children could be referred to testing if CMAM staff received orientation on the importance of referring children to HIV testing. This measure was suggested by staff in facilities which reported lack of commitment and/or appreciation of the importance of testing amongst staff. Further basis of this suggestion was the finding, noted earlier, that at the time of training of staff four years earlier referral of malnourished children to HTC had not been emphasized, neither did reporting tools they were trained on have provision for clients’ sero-status.

## Discussion

This study revealed that only 58% of children enrolled in CMAM in Dowa District were tested for HIV. This is a moderately low delivery. The highest reported percentage delivery of HIV testing in CMAM was 94% as found in Community-based Therapeutic Care Program (now called OTP) [11]; and 91% in NRU [10]. The current study’s finding of moderately low delivery confirms the findings from the national data of 2012 which showed that only 26% children enrolled in CMAM nationally were tested for HIV. Nevertheless, the percentage delivery in Dowa, a small district, was found to be higher than the national average (26%) over the study period, January–December 2012 [8]

We found that intermittent supply of HIV test kits was an important service provider factor affecting delivery of HIV testing in CMAM clients in Dowa district. In the absence of test kits all other efforts by staff, such as motivating guardians to test, are rendered futile. The procedure for replenishment of test kits is that each health facility submits their requirements monthly which are then compiled for the district as a whole. In this regard, it was also found that due to poor coordination among departments in health facilities there are often inaccuracies in the quantities of test kits ordered by the District Health Office, leading to quantities ordered being less than requirements. It was also clear from the study findings that when test kits became available, there was no mechanism for following up on CMAM clients who had previously not been tested during stock outs of test kits. However, the intermittence in supply of kits, as was found in this study, is a reflection of the general shortage and logistical shortcomings in supply of medicines and supplies in the wider government health care system in Malawi [13]. It is therefore clear that stock-out of test kits was indeed a major hindrance to HIV testing in Dowa district.

In addition, where test kits were in short supply, CMAM clients did not receive high priority compared to clients of other programs such as PMTCT. This scenario is entrenched by the finding that some of the test kits are supplied to health facilities by non-governmental organizations (NGOs) specifically for PMTCT. The priority accorded to HIV-focused services over other programs where resources are limited has been found in other studies conducted in Malawi as well [14]. It is clear that throughout the health system in the district there is need for a greater appreciation amongst staff of the importance of providing HIV testing to malnourished children and that HIV testing is an important entry point to other services such as ART which are critical to survival of such children.

The other factor which was found to affect delivery of HIV testing among CMAM clients is shortage of HTC counselors. The shortage of human resource in Malawi’s public health system in general and HIV/AIDS sector in particular, is well documented [15] [16]. Our study found that due to shortage in staff, HTC counselors have an overload of work, as they also perform other duties required of them as health surveillance assistants. This finding supports the findings of the previous study undertaken in Malawi to investigate factors affecting access to HIV testing which revealed the shortage of HTC staff as impeding access of HIV testing [17].

Another factor found to be affecting delivery of HIV testing was the lack of commitment from the staff to refer all malnourished children to HTC, particularly in OTP and SFP. It was revealed that some CMAM clients are not being referred to HTC in the first place and/or those not tested at entry are not followed up during subsequent visits. This action may be due to negative attitudes towards malnourished children and lack of the importance of HIV test in malnutrition. A study conducted among nurses in South Africa reported that staff often have negative attitude towards severely malnourished children [18]. The authors attributed this to lack of knowledge, which was confirmed by the change of attitudes after the training showing that if people know the reasons for certain actions they are likely to cooperate.

In the current study it was revealed that staff were last trained in CMAM about four years prior to this research and by that time there was little, if any, emphasis on referring children for HIV testing. It is worth noting that apart from the obvious benefit of imparting skills on staff, training has indeed been found to be a factor in improving motivation of health workers to do their work [19]. Although training may help to motivate CMAM staff, the lack of commitment and diligence among staff in referring children to HIV testing may also be attributed to low remuneration and the low morale among health workers in the country’s public health system [15].

Related to lack of commitment amongst staff, the study found lack of skills among CMAM staff in motivating clients to test as another factor. This may be attributable to the fact that CMAM staff, who are typically health surveillance assistants by cadre, are not trained in counseling. Neither does standard training in CMAM include counseling. These finding confirms that of a study conducted in Malawi which reported that NRU workers lacked specialized training in HIV counseling [20].

It is therefore important that the CMAM should have staff adequately trained to appreciate the importance of ensuring that all malnourished children are tested so that they can be referred to other services such as ART. Staff would then understand that without these other services treatment of malnourished children living with HIV would not produce desired outcomes.

Differences in the performance of health facilities were also observed in the current study. Health facilities that had a delivery of between 80% and 100% attributed their performance to dedicating enough time and staff to follow up on clients that declined to get tested at the first visit. This action involved repeated counselling to convince clients of the need to undergo testing.

On the other hand, health facilities that performed poorly in terms of delivery of testing, 40%- 62%, reported that this was due to having inadequate staff in the CMAM program who are overwhelmed by a heavy workload. Because of heavy workload, staff were unable to allocate enough time to following up on clients to ensure that they are undergo testing.

It was recommended that in order to improve delivery there was need to deploy more staff in CMAM program with the view that this would reduce workload and enable staff to dedicate enough time to counselling of clients to accept testing.

In the final analysis it should be observed from the findings that absence of an HIV test means that the continuity of care for CMAM clients is hampered, as HTC is an entry point to both PMTCT (for mothers) and ART (for both mothers and children).

## Limitations of the Study

The study may have suffered selection bias due to the small number of facilities studied; as such the findings may not be a reflection of all government-run facilities in the district of Dowa. Nevertheless, considering that the setting in which CMAM is implemented in the district is similar, as observed by the authors, the findings should be a fair reflection of all government-run facilities in the district. This is in addition to the fact that CMAM and HTC are implemented based on the same guidelines and by staff trained under the same regime.

Also, there may have been potential bias in the selection of study respondents as they were selected purposively for being focal persons of either CMAM or HTC. However, this limitation is mitigated by the fact that the respondents (focal persons) provided data which by study design included experiences of other health workers with whom they offer the services in question.

Record keeping of children tested of HIV was found to be poor. This may render the percentage delivery of HIV testing that the study found (58%) to be inaccurate. Nevertheless, the finding that delivery is moderately low is similar to national data, which is what inspired our study as outlined in the ‘problem statement’.

The fact that the district under study was purposively selected means that the findings of this study may not be generalized to other districts offering CMAM in Malawi. Nevertheless, findings of this study may have relevance in other rural districts due to the fact that the national average delivery of HIV testing in CMAM is similar to what was found in this study.

## Conclusions and Recommendations

This study shows the importance of a well-functioning health system in terms of skilled and committed staff and availability of resources needed to implement interventions that will reduce child mortality and achieve Millennium Development Goal 4.

The findings of this study have a number of implications. Firstly, as a way forward, the Ministry of Health and its partners need to allocate more resources into training of more HTC counselors. This may help reduce the workload placed on HTC counsellors who have to perform other duties apart from HIV testing. Secondly, there should also be adequate and equal distribution of HIV test kits across all health programs in Dowa district. There should also be mechanisms for ensuring that when test kits are available clients who may not have been tested during test kit stock-outs are recalled and offered HIV testing. Thirdly, future training of health workers involved in CMAM needs to include other related aspects such as HIV counseling. Adequately trained staff are more likely to appreciate the importance of ensuring that children are tested for HIV and those found to be HIV positive are referred to life saving services such as ART. Fourth, there is need for health policy makers to employ innovative models of HIV testing and counseling as a way of increasing delivery amongst CMAM clients. These innovations may including mechanisms for following up on clients previously not tested for reasons such as those found in this study.
